# Stable superhydrophobic surface of hierarchical carbon nanotubes on Si micropillar arrays

**DOI:** 10.1186/1556-276X-8-412

**Published:** 2013-10-07

**Authors:** Shaoqing He, Jinquan Wei, Haifan Wang, Deshun Sun, Zhaohui Yao, Chengsong Fu, Ruiqiao Xu, Yi Jia, Hongwei Zhu, Kunlin Wang, Dehai Wu

**Affiliations:** 1 Key Lab for Advanced Materials Processing Technology of Education Ministry, School of Materials Science and Engineering, Tsinghua University, Beijing 100084, People's Republic of China; 2 School of Mechatronic Engineering, China University of Mining and Technology, Beijing 100083, People's Republic of China; 3 School of Aerospace, Tsinghua University, Beijing 100084, People's Republic of China

**Keywords:** Carbon nanotube, Hierarchical architecture, Superhydrophobic surface

## Abstract

It is of great importance to construct a stable superhydrophobic surface with low sliding angle (SA) for various applications. We used hydrophobic carbon nanotubes (CNTs) to construct the superhydrophobic hierarchical architecture of CNTs on silicon micropillar array (CNTs/Si-μp), which have a large contact angle of 153° to 155° and an ultralow SA of 3° to 5°. Small water droplets with a volume larger than 0.3 μL can slide on the CNTs/Si-μp with a tilted angle of approximately 5°. The CNTs growing on planar Si wafer lose their superhydrophobic properties after exposing to tiny water droplets. However, the CNTs/Si-μp still show superhydrophobic properties even after wetting using tiny water droplets. The CNTs/Si-μp still have a hierarchical structure after wetting, resulting in a stable superhydrophobic surface.

## Background

Interfacial interaction between liquid and solid is of great importance for materials in various applications, such as absorption, adhesion, lubrication, and transference. Due to easy deformation of liquid, large droplets slide on a solid surface easier than the small ones. The mobility of droplets depends not only on the properties and size of liquid but also on the surface state of solid [1]. Superhydrophobic surfaces which have a static contact angle (CA) larger than 150° [2] are desired in collecting and delivering tiny water droplets in some cases [3,4]. Various approaches have been established to construct superhydrophobic surfaces, such as coating with hydrophobic materials [5-7], increasing roughness [8,9], and fabricating hierarchical micro/nanoarchitectures [10-12]. Interfacial interaction hinders the motion of stationary water droplets on a solid surface, resulting in CA hysteresis. The CA hysteresis on a superhydrophobic surface might result from high adhesive force and absorption [13,14], which implies that it is not easy for tiny water droplets to move on such surface. Up to now, most of the research on superhydrophobic surface focused on measuring the CAs and sliding angles (SAs) of water droplets with a volume not smaller than 2 μL (approximately 1.6 mm in diameter). However, we often observe water droplets with a volume lower than 2 μL, such as fog droplets, existing or sliding on a solid surface in nature. There is a need to reveal the interfacial interaction between superhydrophobic surface and tiny water droplets.

Generally, pristine carbon nanotubes (CNTs) are hydrophobic materials, which have also been used to construct a superhydrophobic surface [15,16]. By making micropatterns, the hydrophobicity of a CNT surface is further enhanced. The CA between water and CNT pattern is usually larger than 150°, but the SA is also large (usually larger than 30°) [17,18]. However, the superhydrophobic CNT forest might also absorb water, resulting in collapsing into cellular foams when water evaporates from interstices of nanotubes [19]. After wetting, the CNT forest might lose its superhydrophobic properties. It needs to construct a stable and durable superhydrophobic surface even wetted by vapor or tiny water droplets. Here, we fabricate the superhydrophobic hierarchical architecture of CNTs on Si micropillar array (CNTs/Si-μp) with large CA and ultralow SA. The CNTs/Si-μp show a durable superhydrophobic surface even after wetting using tiny water droplets.

## Methods

**Figure 1:**
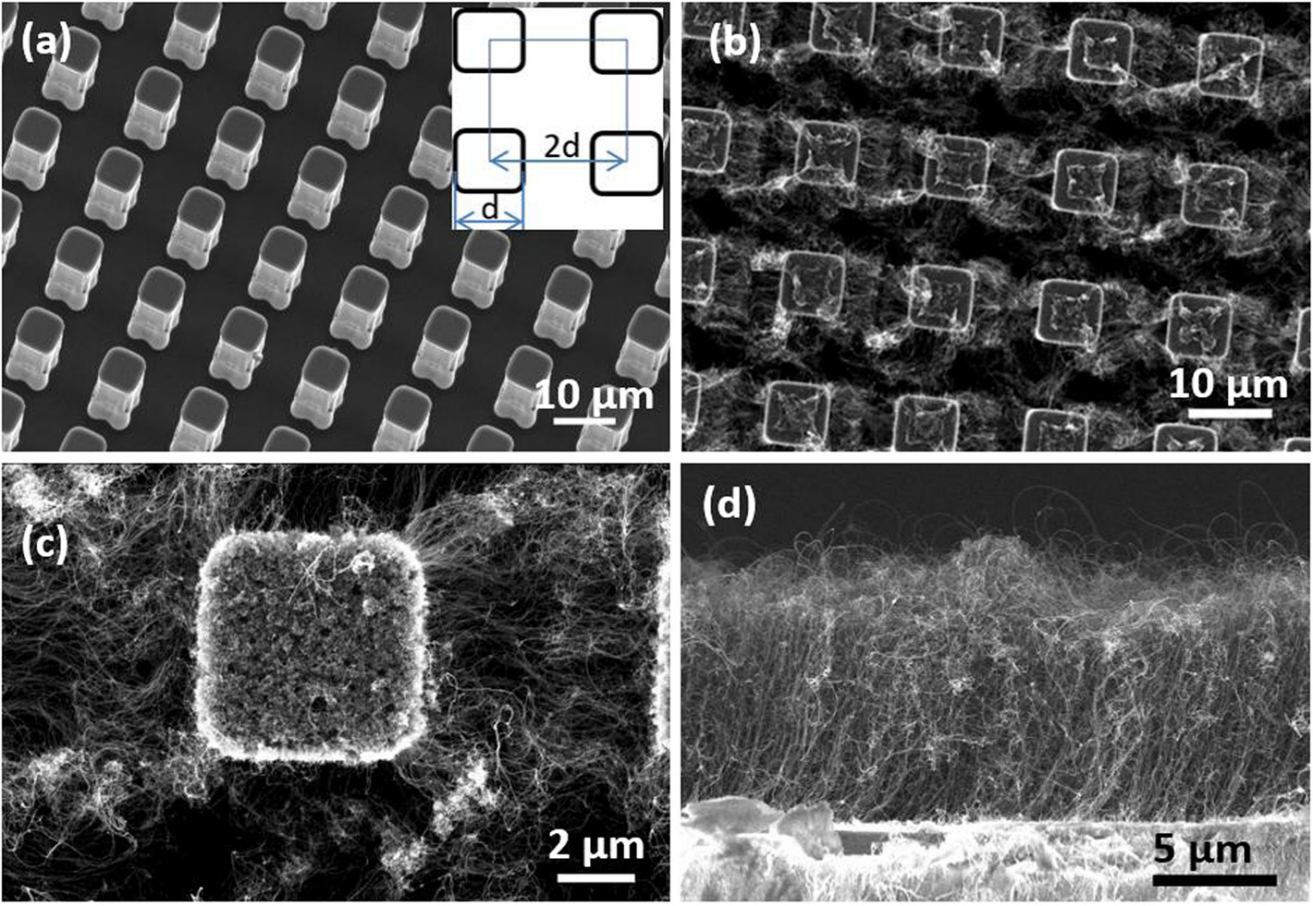
**SEM characterization of various samples. (a)** Si micropillar array. **(b)** Hierarchical architecture of CNTs/Si-μp. **(c)** Connection between a Si micropillar and CNT forests. **(d)** CNT forest growing on a planar Si wafer.

Si micropillar (Si-μp) arrays with defined squares (see Figure 1a, inset) were etched from a Si (100) wafer by ultraviolet lithography (UVL) and deep reactive-ion etching (DRIE) in sulfur hexafluoride (SF_6_) and perfluoro-2-butene (C_4_F_8_). The height of the Si-μp was controlled by etching time. A standard cleaning process developed by the company Radio Corporation of America (RCA) was carried out to eliminate residual metal and organic species followed by removing Si oxide in a buffered HF solution. The Si micropillar arrays and planar Si wafer were coated with a thin layer of aluminum (10 nm) using an e-beam evaporator for CNT growth. CNTs were grown by floating chemical vapor deposition method, using xylene as carbon source, ferrocene as catalyst precursor, and a mixture of Ar and H_2_ as carrier gas, according to our previous report [20]. During the growth of CNTs, the ferrocene/xylene solution (20 mg/mL) was fed into the reactor at a rate of 0.2 mL/min, and Ar and H_2_ were fed at 400 and 50 sccm, respectively.

The samples were characterized using a scanning electron microscope (SEM). The CA and SA were measured using a contact angle goniometer (Rame-hart 300, Rame-hart Instrument Co., Succasunna, NJ, USA). The CNT samples mounted on an inclined substrate with a slope of 5° were exposed to tiny water droplets (50 to 500 μm in diameter) generated from a nebulizer. The tiny water droplets on the CNT forest were observed using a stereomicroscope (Stemi 2000, Carl Zeiss, Inc., Oberkochen, Germany).

## Results and discussion

The Si-μp arrays used in the experiment have a square shape with spacing equal to the dimension. The area fraction of the Si-μp arrays is *f* = 0.25 (*f* = *a*^2^ / (*a* + *b*)^2^, where *a* is the dimension of micropillars and *b* is the spacing between the neighboring pillars). Figure 1a is a tilted-view SEM image of the Si-μp array with a dimension of 8 μm, showing well-defined pillars with a smooth surface. The height of the micropillar is about 15 μm.

Figure 1b is a SEM image of the CNT forest growing on Si-μp arrays, showing the hierarchical architecture of CNTs/Si-μp. The forest comprises a large amount of loose CNTs. Figure 1c is a SEM image of a single Si-μp with mutually orthogonal CNT forests. The forests growing on two neighbor micropillars already join together after 6-min CNT growth. For comparison, we prepared the CNT forest on planar Si wafers (CNTs/Si) using the same growing parameters. Some CNTs extruding from the forest are observed during SEM examination, forming a rough surface (see Figure 1d). The density of CNTs within the forest growing on the planar Si is similar to that growing on the Si-μp arrays. The height of the forest is approximately 10 μm after 6-min CNT growth.

**Figure 2:**
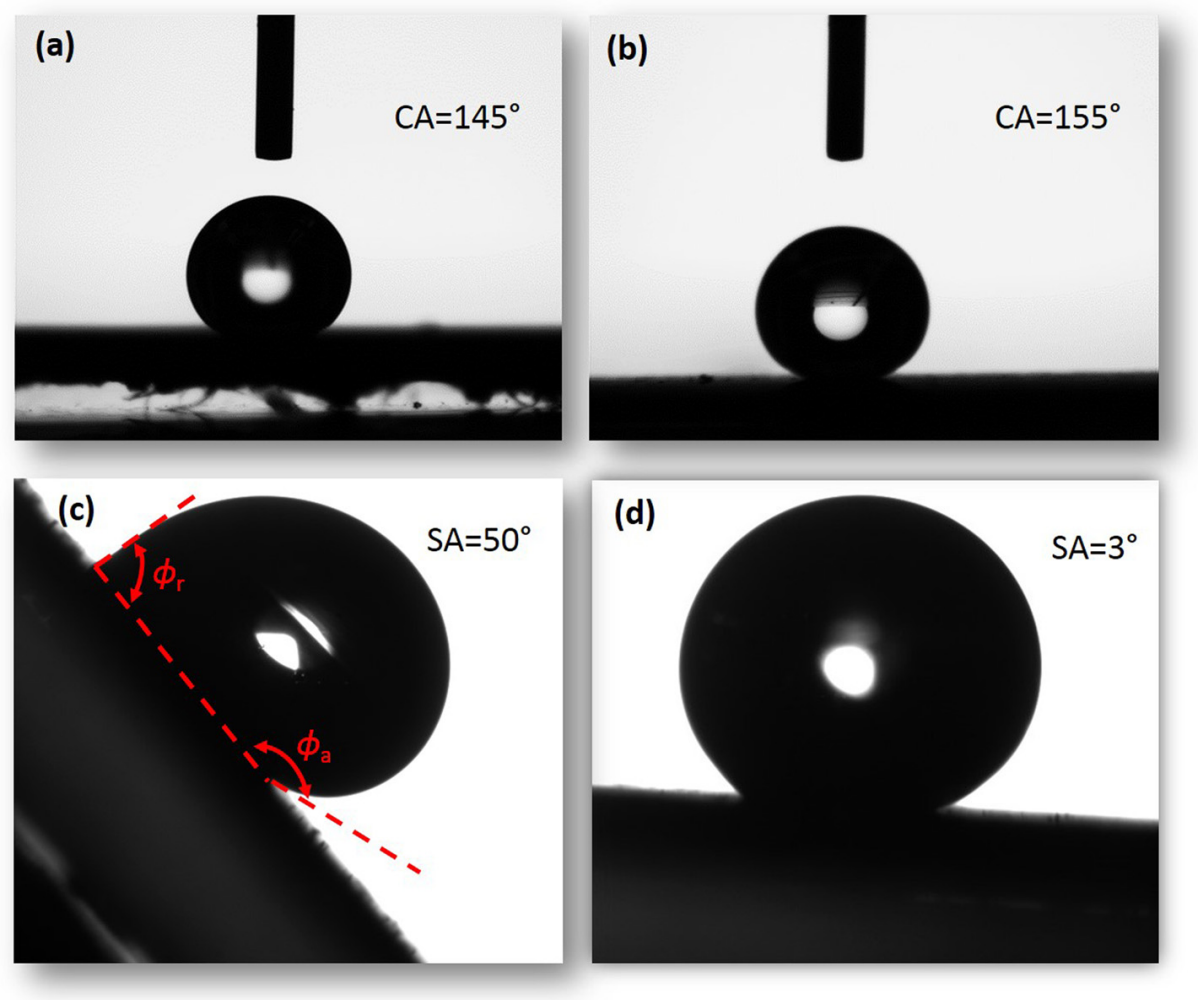
**Contact and sliding angles of water droplets on CNTs/Si and CNTs/Si-μp.** Contact angles of water droplets on **(a)** CNTs/Si and **(b)** CNTs/Si-μp. Sliding angles of water droplets on **(c)** CNTs/Si and **(d)** CNTs/Si-μp. The volume of water droplets is 7 μL.

The static CAs of water on CNTs/Si and CNTs/Si-μp are measured using 7 μL of (approximately 2.4 mm in diameter) water droplets. Figure 2a shows an image of a water droplet on the CNT forest with 8 μm in height growing on Si. The CA between water droplet and CNTs/Si is 145°, showing the hydrophobic surface of CNTs/Si. Table 1 gives the CA of water on CNTs/Si with different CNT heights. It shows that the CA increases as the CNT height increases. For the 15-μm CNTs/Si surface, the CA is about 150°, showing a superhydrophobic property according the static CA criteria [2].

**Table 1:** CA and SA of water droplets (7 μL) on various CNT surfaces

**Sample**	**5-μm CNTs/Si (deg)**	**8-μm CNTs/Si (deg)**	**10-μm CNTs/Si (deg)**	**15-μm CNTs/Si (deg)**	**CNTs/Si-μp, 16-μm Si pillar (deg)**	**CNTs/Si-μp, 8-μm Si pillar (deg)**
CA	143	145	147	150	153	155
SA	55	50	40	40	5	3

Figure 2b shows the CA between water droplet and CNTs/Si-μp with a dimension of 16 μm. The CA of the CNTs/Si-μp surface is 155°, showing the superhydrophobic surface of hierarchical CNTs/Si-μp. There are two kinds of air cavities in the hierarchical CNTs/Si-μp: air between Si micropillars and air between CNTs. The CA of water droplets on CNTs/Si-μp can be expressed by Cassie's law:

$$\cos\theta_{\mathrm{CNTs}/\mathrm{Si}‒\mathrm{\mu p}}=f_{\mathrm{CNTs}}\cos\theta_{\mathrm{CNTs}}+f_{\mathrm{Si}‒\mathrm{\mu p}}\cos\theta_{\mathrm{Si}‒\mathrm{\mu p}},$$

where *f*_*x*_ is the areal fraction of *x* and *θ*_*x*_ is the contact angle of water with surface *x*. Because the Si micropillars are covered by CNTs, the CA of CNTs/Si-μp is larger than that of CNTs/Si. The CA increases slightly from 153° to 155° when the dimension of Si micropillars reduces from 16 to 8 μm (see Table 1).

The mobility of water droplets on a CNT forest surface was investigated by measuring the SA. Figure 2c shows an image of a water droplet which begins to slide on an inclined CNTs/Si surface with a slope of approximately 50°. It shows a significant CA hysteresis of approximately 77° with an advancing angle of *Φ*_a_ = 163° and a receding angle of *Φ*_r_ = 86°. The SA of CNTs/Si varies from 40° to 50° according to the height of the CNT forest (see Table 1). The large CA hysteresis implies that it is hard for water droplets to slide on the CNTs/Si surface. Figure 2d shows an optical image of a water droplet sliding on CNTs/Si-μp. The water droplet on hierarchical CNTs/Si-μp has no evident hysteresis with an ultralow SA of 3° to 5°. The ultralow SA implies that water droplets are easy to slide on the CNTs/Si-μp surface.

**Figure 3:**
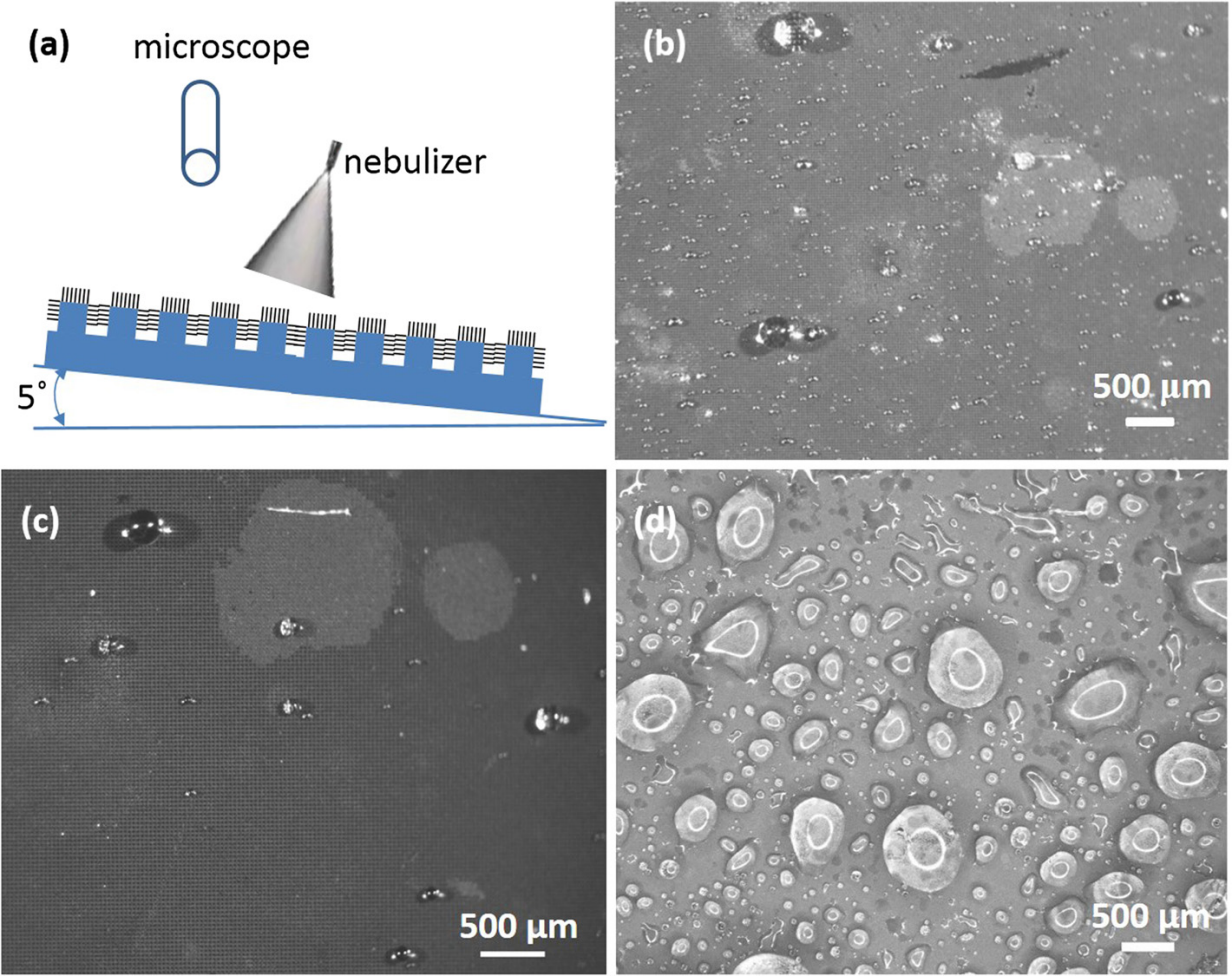
**Representation of water droplets in different conditions. (a)** Schematic figure of tiny water droplets sprayed from a nebulizer. **(b)** Tiny water droplets on CNTs/Si-μp surface. **(c)** Water droplets on CNTs/Si-μp after three time tests. **(d)** Water droplets on CNTs/Si surface.

We further reveal the behaviors of tiny water droplets on CNTs/Si and CNTs/Si-μp. Because the SA of CNTs/Si-μp is 3° to 5°, we mounted CNT samples on an inclined substrate with a slope of 5°. The CNT forest is then exposed under tiny water droplets with a diameter of 50 to 500 μm sprayed from a nebulizer (see Figure 3a). The situations of tiny water droplets are quite different from those of large droplets used in SA measurement. Some of the tiny droplets might join into larger ones and slide down on the CNTs/Si-μp, while some of them might stick on the CNTs/Si-μp surface. The water droplets sticking on the CNTs/Si-μp surface have a round shape (see Figure 3b). The largest water droplets we observed on the CNTs/Si-μp surface have a diameter less than 0.8 mm (approximately 0.27 μL), which implies that water droplets larger than 0.3 μL might slide on the CNTs/Si-μp surface with a tilted angle of 5°. It indicates that the hierarchical CNTs/Si-μp can be used to collect tiny water droplets. Most of the tiny water droplets are absorbed by the CNT forest eventually within 10 min. The CNTs/Si-μp surface is thus wetted by exposing under tiny water droplets for a long time. However, the wetted CNTs/Si-μp surface still shows superhydrophobic behaviors after it dries up. Figure 3c shows an image of the CNTs/Si-μp exposed under tiny water droplets after three time tests. The shape of water droplets is quite similar to those in Figure 3b, which indicates that the CNTs/Si-μp surface still shows hydrophobic properties after wetting using the tiny water droplets.

For comparison, we provide a microscopic image of CNTs/Si exposed under nebulizer fogs in Figure 3d. It is not easy for water droplets to slide on the CNTs/Si surface due to large SA. Some water droplets sprayed into CNTs/Si disperse into the cavities of the CNT forest, making the wetting surface of the CNTs and some tiny water droplets gather into large drops. The large water droplets on the CNTs/Si surface deform into irregular shapes due to wetting, which are quite different from those on the CNTs/Si-μp. The water droplets we observed on the CNTs/Si surface have a diameter above 5 mm (approximately 52 μL).

**Figure 4:**
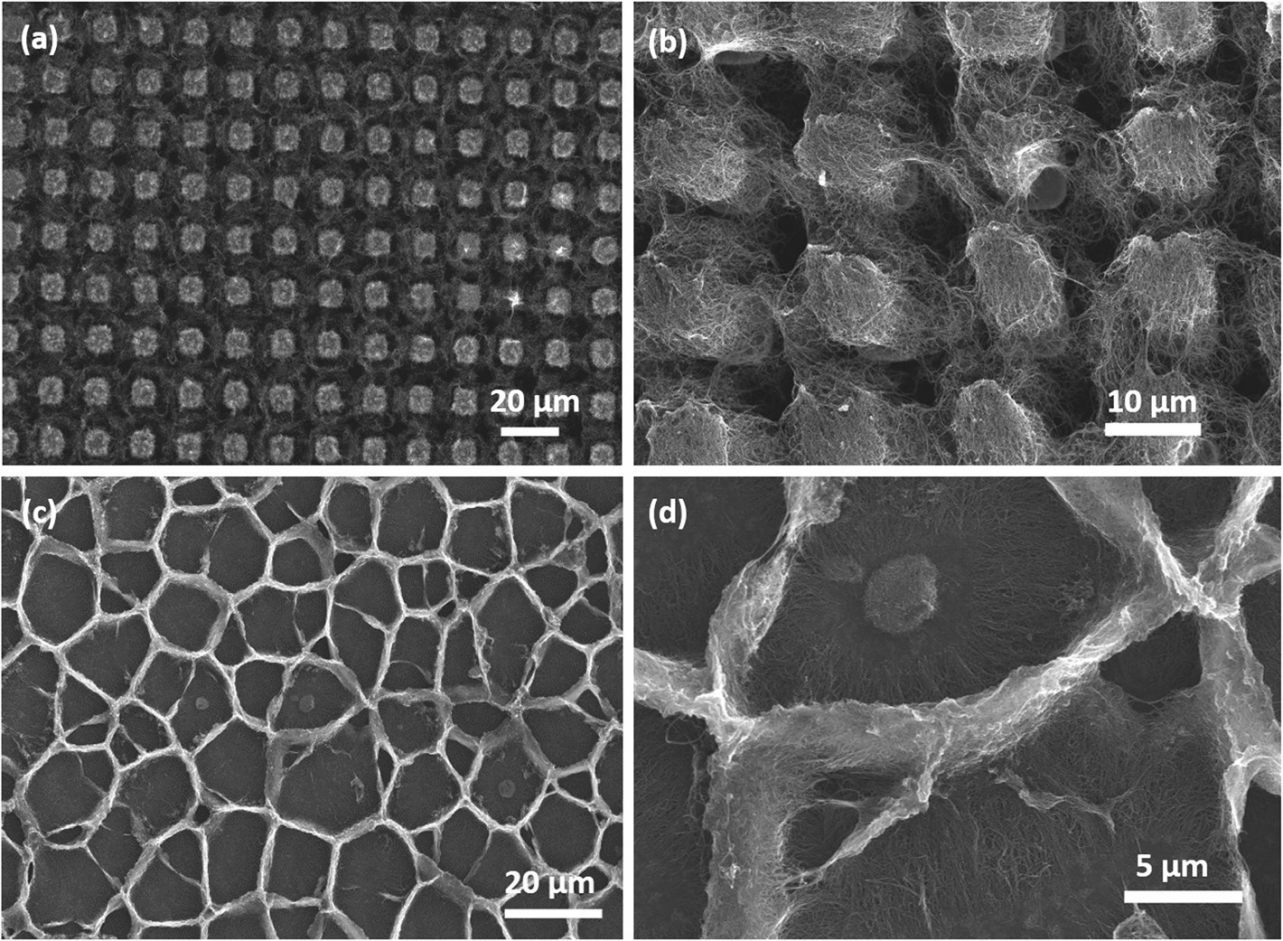
**SEM images of CNTs/Si-μp and CNTs/Si after wetting. (a)** Low- and **(b)** high-magnification SEM images of CNTs/Si-μp after wetting using nebulizer droplets. **(c)** Low- and **(d)** high-magnification SEM images of CNTs/Si after wetting using nebulizer droplets.

In our experiments, the CNT forest, no matter growing on planar Si wafer or Si micropillars, might absorb tiny water droplets. The CNTs/Si-μp still have superhydrophobic properties after adsorbing water and drying. In contrast, the CNTs/Si lose their superhydrophobic properties. Figure 4 shows SEM images of the CNT forest after wetting using tiny water droplets. It is clear that the CNT forest shrinks driven by capillarity force after wetting, but the CNTs still suspend among the Si micropillars (Figure 4a,b). Although the air cavities within CNTs might reduce significantly, the air cavities between Si micropillars are maintained. The CNTs/Si-μp still have a hierarchical structure after drying and thus show hydrophobic properties. For the CNTs growing on planar Si wafer, vertical-standing CNTs were destroyed and form a cellular structure on Si wafer (Figure 4c,d), which is similar to a recent report [19]. The air cavities within CNTs are eliminated, so the CNT forest on planar Si wafer loses its superhydrophobic properties.

## Conclusions

In summary, the hierarchical architecture of CNTs/Si-μp has a superhydrophobic surface with large CA and ultralow SA of only 3° to 5°. Tiny water droplets larger than 0.3 μL can slide on CNTs/Si-μp with a tilted angle of 5°, showing a high capacity of collecting water droplets. After wetting using tiny water droplets, the CNT forest growing on planar Si wafer loses its superhydrophobic properties, but the CNTs/Si-μp still have a superhydrophobic surface because they still have a hierarchical structure. The CNTs/Si-μp show stable superhydrophobic properties.

## Competing interests

The authors declare that they have no competing interests.

## Authors' contributions

SQH, JQW, HFW, and DSS performed the experiments and fabricating the hierarchical structure. JQW, ZHY, and CSF coordinated the project. RQX and YJ performed the SEM measurement. HWZ, KLW, and DHW discussed the results. SQH and JQW drafted the paper. All authors read and approved the final manuscript.
